# Protective Effects of Andrographolide Analogue AL-1 on ROS-Induced RIN-mβ Cell Death by Inducing ROS Generation

**DOI:** 10.1371/journal.pone.0063656

**Published:** 2013-06-04

**Authors:** Guang-Rong Yan, Hui-Hua Zhou, Yang Wang, Yin Zhong, Zi-Lu Tan, Yuqiang Wang, Qing-Yu He

**Affiliations:** 1 Key Laboratory of Functional Protein Research of Guangdong Higher Education Institutes, Institute of Life and Health Engineering, College of Life Science and Technology, Jinan University, Guangzhou, China; 2 Institute of New Drug Research, College of Pharmacy, Jinan University, Guangzhou, China; Osaka University Graduate School of Medicine, Japan

## Abstract

Oxidative stress is considered to be a major factor contributing to pathogenesis and progression of many diseases. A novel andrographolide-lipoic acid conjugate (AL-1) could protect pancreatic β-cells from reactive oxygen species (ROS)-induced oxidative injury. However, its protective mechanism is still unclear. In this work, we used proteomics to identify AL-1-regulated proteins in β-cells and found that 13 of the 71 proteins regulated by AL-1 were closely associated with antioxidation. These differential proteins were mainly involved in the ERK1/2 and AKT1 signaling pathways. Functional investigation demonstrated that AL-1 exerted its protective effects on H_2_O_2_-induced cell death of β-cells by generating NADPH oxidase-dependent ROS to activate ERK1/2 and AKT1 signaling pathways. As a consequence, the expressions of antioxidant proteins including Trx1, Prx1 and Prx5, and anti-apoptotic proteins including PDCD6IP, prohibitin, galectin-1 and HSP were upregulated. AL-1 probably worked as a “vaccinum” to activate the cellular antioxidant system by inducing the generation of low concentration ROS which then reciprocally protected β-cells from oxidative damage caused by high-level ROS from H_2_O_2_. To the best of our knowledge, this is the first comprehensive proteomic analysis illustrating a novel molecular mechanism for the protective effects of antioxidants on β-cells from H_2_O_2_-induced cell death.

## Introduction

Reactive oxygen species (ROS) are chemically high-reactive oxygen-based molecules that play a key role in many physiological and pathophysiological processes. Its intracellular concentration was regulated by both free radical production and antioxidant defenses [1]. In physiologic concentrations, endogenous ROS are essential signaling intermediates that regulate cell survival, growth, metabolism and motility [2], [3]. Enhanced intracellular ROS after diverse stimuli could cause chronic oxidative stress and adverse effects. Accumulated ROS can directly injure cells and induce cell apoptosis and necrosis through damaging macromolecules, membranes and DNA [1].

The production and accumulation of ROS have been considered as a major cause of the pathogenesis and development of many diseases. For example, Hyperglycemia-generated ROS induces pancreatic β-cell dysfunction found in diabetes, playing a key role in the pathogenesis and progression of diabetes and diabetic complications [4]. ROS contributes to skin aging, skin disorders, and skin diseases [5]. ROS accumulation has been implicated in the pathogenesis of numerous cardiovascular diseases and has been linked to cardiomyocyte hypertrophy, myocardial remodeling, and heart failure [6]. Oxidative stress induced by ROS is also considered to be an important part of the etiology of atherosclerosis [7]; and ROS-induced oxygen toxicity is known to be one of the major contributors to bronchopulmonary dysplasia [8]. ROS-mediated oxidative stress is involved in the neuropathological processes by inducing neuronal cell death such as Parkinson's disease, Alzheimer's disease, Huntington's disease (HD), amyotrophic lateral sclerosis (ALS), ischemia/reperfusion, schizophrenia, drug abuse, tardive dyskinesia, seizure disorders, manganese neurotoxicity, as well as the aging brain [9].

One of the plausible ways to prevent ROS-mediated cellular injury is dietary or pharmaceutical augmentation of endogenous antioxidant defense capacity. Convincing data has been accumulated in the treatment of oxidative stress-induced cell injury using natural products or extracts from plants [10]. For example, isoflavone has been shown to significantly decrease post menopause-related cardiovascular diseases [11]. Both antioxidant nutrients and antioxidant phytochemicals could alleviate diabetes and diabetic complications by suppressing oxidative stress-induced β-cell apoptosis and dysfunction [12]–[14]. Therefore, pharmacological interventions targeting ROS has become a focus in biomedical research.

Andrographolide-lipoic acid conjugate (AL-1) is a new chemical entity derived by covalently linking andrographolide (andro) with lipoic acid (LA), two molecules previously shown to have anti-diabetes property [15]–[17]. High dose AL-1 exerts its anti-cancer cytotoxicity through a ROS-dependent DNA damage and mitochondria-mediated apoptosis mechanism in human leukemia K562 cells [18]. Interestingly, our previous studies also showed that low dose AL-1 could decrease blood glucose, increase insulin secretion, and protect the apoptosis of β-cells in alloxan-induced diabetic mouse model [17]. The pretreatment of RIN-mβ cells with AL-1 effectively prevented ROS-induced cell death in H_2_O_2_-induced β-cell oxidative stress model [17]. However, the protective mechanism of AL-1 on pancreatic β-cells is still poorly understood. In this work, we firstly used proteomics technology to identify AL-1-regulated proteins in this model, and then performed functional studies to reveal that AL-1 activated ERK1/2 and AKT1 signaling pathways and subsequently upregulated the expression of antioxidation proteins to prevent pancreatic β-cells from death via inducing the generation of low concentration ROS. The current study provides new insights into the protective mechanism of AL-1 on β-cells.

## Results

### AL-1 attenuated H_2_O_2_-induced RIN-mβ cell death

**Figure 1:**
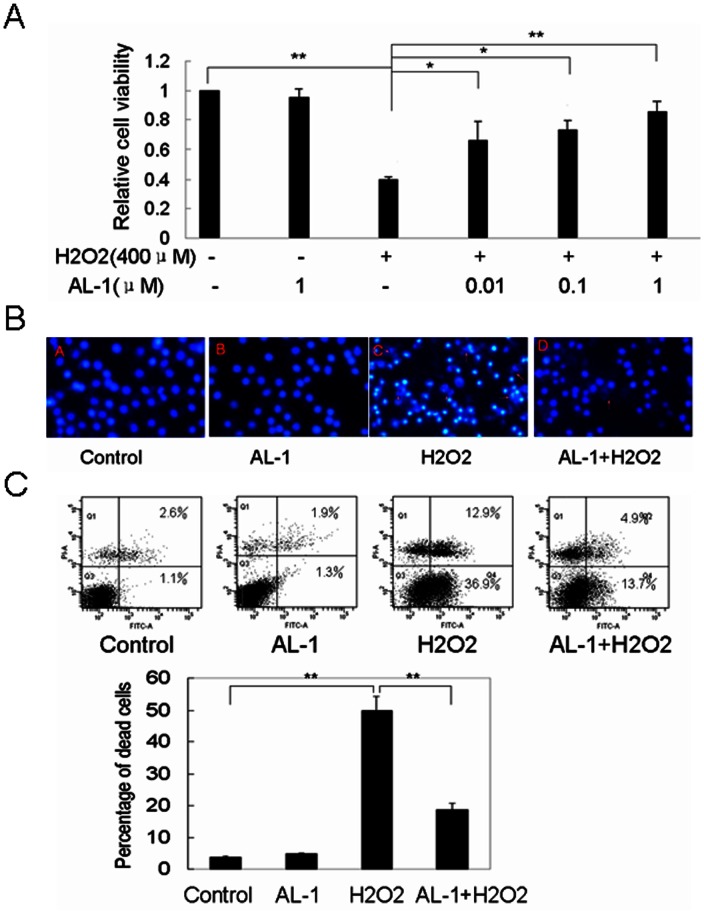
AL-1 attenuated H_2_O_2_-induced RIN-mβ cell death. (A) Effect of AL-1 on H_2_O_2_-induced cell viability. The RIN-mβ cells were pretreated with different concentrations (0.01, 0.1, 1 μM) of AL-1 prior to 400 μM H_2_O_2_ exposure for 4 h. The cell viability was measured by MTT assay. (B) Flow cytometric analysis for the AL-1 protection of RIN-mβ cells against H_2_O_2_-induced death. The RIN-mβ cells were treated with 0.1 μM AL-1, 400 μM H_2_O_2_, or 0.1 μM AL-1 for 1 h prior to 400 μM H_2_O_2_. The number of apoptotic cells was measured by flow cytometer.

To determine the protective effects of AL-1 on H_2_O_2_-induced cell death, RIN-mβ cells were pretreated with different concentrations (0.01, 0.1, 1 μM) of AL-1 prior to 400 μM H_2_O_2_ exposure for 4 h. MTT assay showed that the number of the surviving cells was increased by AL-1 in a dose-dependent manner as compared to the treatment with H_2_O_2_ alone, while the AL-1 itself had no effect on the cell death (Fig. 1A). Also the cells were pretreated with 0.1 μM AL-1 for the different time (0, 0.5, 1, 2, 4, 8, 12, 24 h) prior to 400 μM H_2_O_2_ exposure for 4 h, MTT assay demonstrated that AL-1 exhibited the protective effect against H_2_O_2_-induced cell death when its pretreatment time was less than 8 h (Fig. S1). These observations suggested that AL-1 could attenuate H_2_O_2_-induced cell death. To exclude a direct protective effect of AL-1, the cells were co-treated with the different concentrations of AL-1 (0, 0.01, 0.1, 1 μM) plus 400 μM H_2_O_2_ for 4 h, our results showed that the cell viability was not significantly different as compared to the treatment with H_2_O_2_ alone, suggesting that AL-1 had not direct protective effect on the high dose H_2_O_2_-induced cell death (Fig. S2). Hoechst 33258 staining demonstrated massive nuclear condensation, a typical morphology characteristic of apoptotic cells/bodies [19], in cells exposed to H_2_O_2_, while the nuclear condensation significantly decreased in AL-1-pretreated cells (Fig. 1B). The protective effects of AL-1 on H_2_O_2_-induced cell death were further investigated by flow cytometric analysis. The percentage (18.6%) of cell death in RIN-mβ cells pretreated with 0.1 μM AL-1 for 1 h prior to 400 μM H_2_O_2_ exposure for 4 h was substantially lower than that (49.8%) in the control cells treated with 400 μM H_2_O_2_ alone for 4 h (Fig. 1C). Taken together, these observations demonstrated that AL-1 could attenuate H_2_O_2_-induced cell death in RIN-mβ cells.

### Proteomic profiles regulated by AL-1

**Figure 2:**
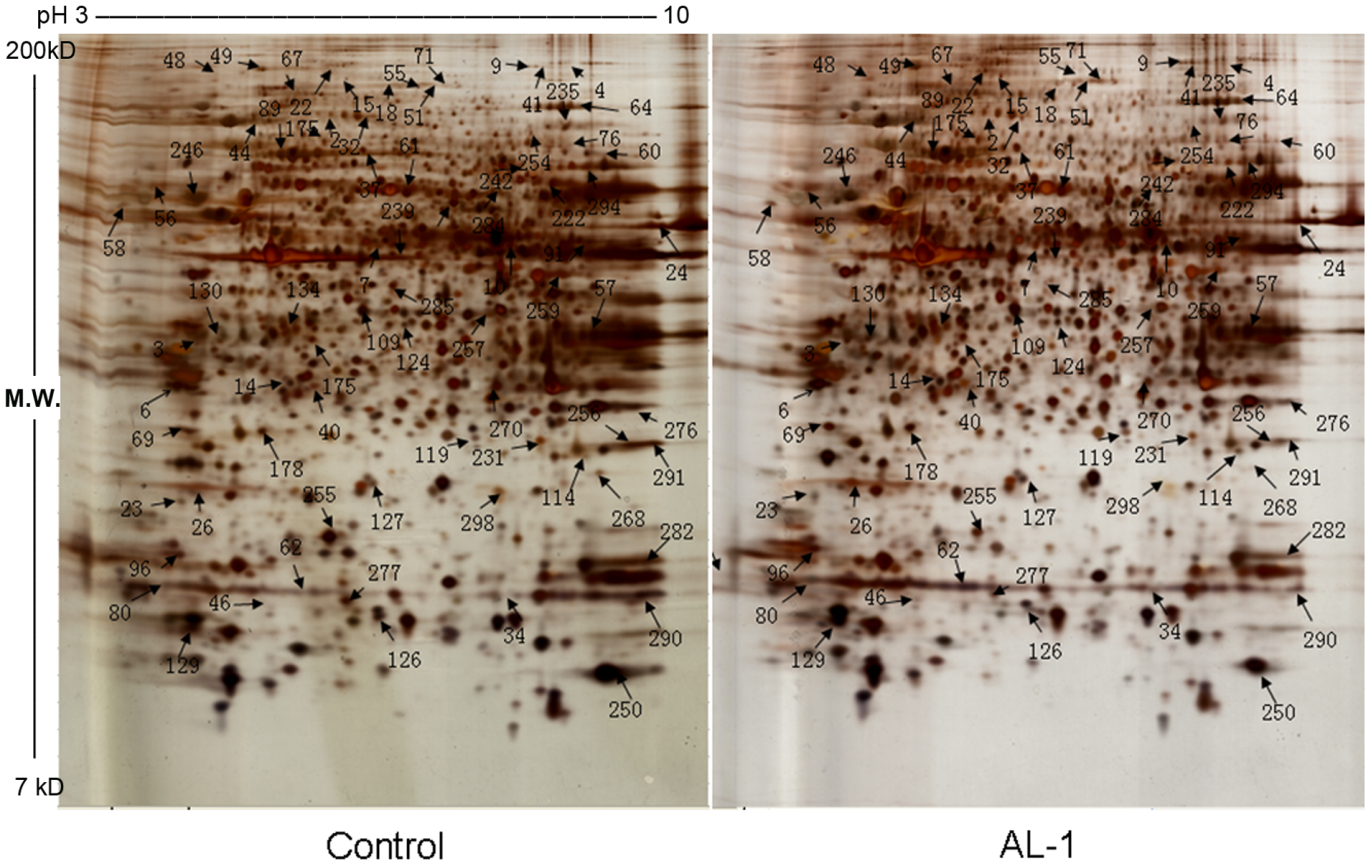
Image overview of 2-DE gels for the proteins extracted from RIN-mβ cells treated with and without AL-1 for 1 h. 150 μg proteins from RIN-mβ cells treated with and without AL-1 were separated by 2-DE, and the gels were stained with sliver. Shown are the representative results from three independent experiments.

Total proteins extracted from RIN-mβ cells treated with and without 0.1 μM AL-1 for 1 h were separated on 2-DE to compare the differential proteins regulated by AL-1 (Fig. 2). Altogether, Over 1000 protein spots were detected in each gel by using ImageMaster software. Protein spots altered greater than 1.5-fold in spot intensity and observed in three replicate gels from three independent experiments were scored and subjected to MS analysis. This allowed us to identify 71 proteins from 105 reproducible differential spots, including 52 increases and 19 decreases in AL-1 treatment gels (Table S1).

As a comparison, 2-DE was respectively performed to separate total proteins from RIN-mβ cells treated with 400 μM H_2_O_2_ alone for 4 h and pretreated with 0.1 μM AL-1 for 1 h followed by 400 μM H_2_O_2_ treatment for 4 h (Fig. S3). In total, 21 proteins, including 14 increases and 7 decreases in their expression, were identified in the gels with AL-1+ H_2_O_2_ treatment as compared to the H_2_O_2_-only treatment (Table S2). Nine of the 21 proteins have been proven to be involved in the regulation of apoptosis including PDCD6IP, hnRNP H, prohibitin, galectin-1, NuMA, RHO-GDI1, HSP9, HSP5a and HSP60. These proteins were effector proteins of AL-1 in the process of AL-1 attenuated RIN-mβ apoptosis induced by H_2_O_2_.

**Figure 3:**
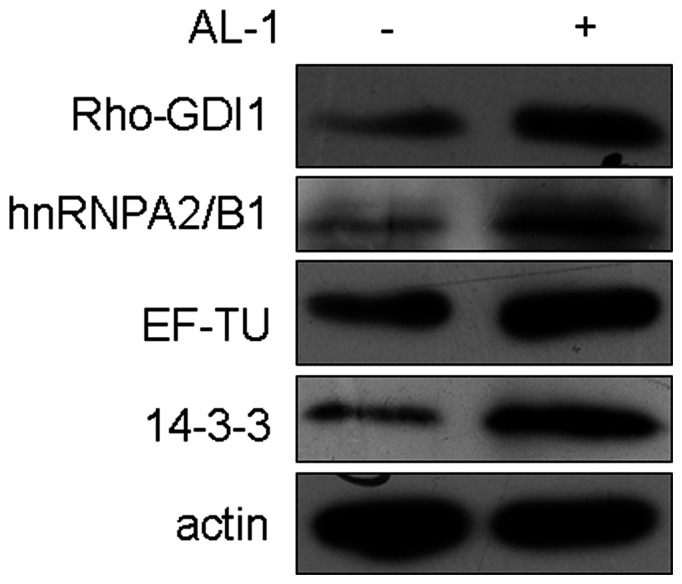
Validation of the proteomics-based quantitation by Western blotting of representative proteins. The proteins were detected by Western blotting analysis with anti-Rho-GDI 1, hnRNPA2/B1, EF-TU, 14-3-3ξ, respectively. The change trends were consistent with the proteomics data.

To validate these proteomic data, representative proteins with differential expressions were analyzed by Western blotting. As shown in Figure 3, the Western blotting results for all the selected proteins were consistent with the change trends of the corresponding proteomic quantitative ratios.

### Antioxidation proteins regulated by AL-1

**Figure 4:**
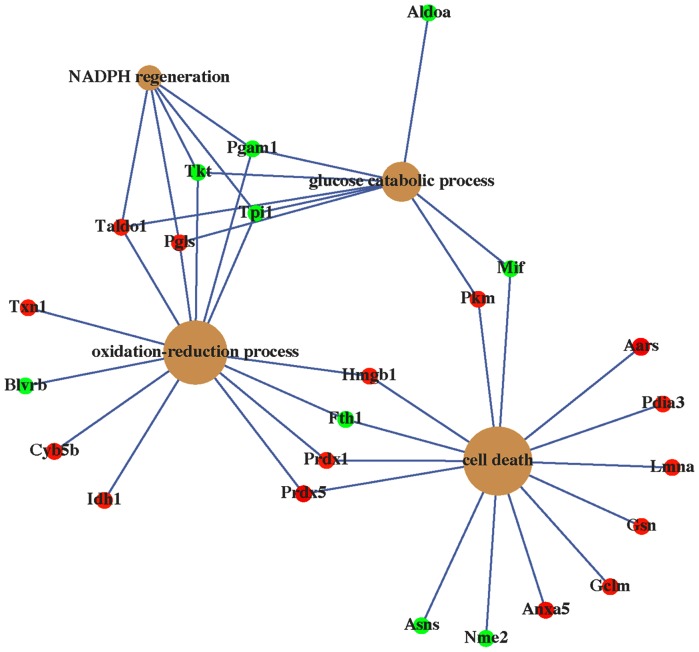
Concept-gene networks of enriched biological processes of AL-1-regulated proteins that were analyzed by Bioconductor package clusterProfiler and visualized by GeneAnswers program.

Interestingly, among the 71 AL-1-regulated differences, 13 proteins including thioredoxin 1 (Trx 1), peroxiredoxin 1 (Prx 1), peroxiredoxin 5 (Prx 5), glutamate-cysteine ligase, 14-3-3ξ, RHO-GDI1, DJ-1, and heat shock protein (HSP) family such as Hspa8, Hspa14, Hyou1, Hsph1 and Hsp90ab1 were known to be associated with anti-oxidation (Table 1). The enrichment of the antioxidant proteins suggested that AL-1 exerted its protective effect against H_2_O_2_-induced cell death possibly by regulating anti-oxidant proteins. Gene Ontology (GO) annotation and Ingenuity Pathway Analysis (IPA) were used to further analyze these AL-1-regulated proteins in terms of the biological process (BP) and involved signaling pathways. GO annotation showed that these differential proteins were mainly categorized into four significant groups according to their biological processes, including oxidation-reduction process, NADPH regeneration, glucose catabolic process and cell death by Bioconductor package clusterProfiler and GeneAnswers program (Fig. 4) [20], [21]. IPA analysis demonstrated that the 71 proteins were involved in five canonical pathways, including NRF2-mediated oxidative stress response, glycolysis, pentose phosphate pathway, pentose phosphate pathway (non-oxidative branch), sucrose degradation (mammalian), belonging to the two groups of oxidative stress response and carbohydrate metabolism. As shown in Figure 4, there is a crosstalk between the two signaling pathways of oxidative stress response and carbohydrate metabolism. We therefore speculate that AL-1 exerts its protective effect against H_2_O_2_-induced cell death by inducing oxidative stress response and upregulating anti-oxidant proteins.

**Table 1:** 13 Proteins associated with antioxidation were identified to be regulated by AL-1.

Spot No.	Accession No.	Protein Name	Gene Name	Peptides	Coverage (%)	Protein Score	Ratio ± S.D.
14	IPI00114329	Glutamatecysteine ligase regulatory subunit	GCLM	8	35	148	4.41±0.33
6	IPI00116498	14-3-3 protein zeta/delta	YWHAZ	24	62	225	5.27±0.38
129	IPI00226993	Thioredoxin	TXN1	9	57	128	1.76±0.15
178	IPI00322312	Rho GDP-dissociation inhibitor 1	ARHGDIA	11	43	181	1.83±0.18
37	IPI00119066	Isoform 1 of Heat shock 70 kDa protein 14	HSPA14	18	44	114	2.87±0.28
127	IPI00116450	Isoform 1 of Core-binding factor subunit beta	CBFB	13	54	100	1.63±0.15
175	IPI00153740	Activator of 90 kDa heat shock protein ATPase homolog 1	AHSA1	14	42	200	1.56±0.05
49	IPI00123342	Hypoxia up-regulated protein 1 precursor	HYOU1	27	32	225	2.64±0.26
89	IPI00323357	Hspa8 Heat shock cognate 71 kDa protein	HSPA8	30	54	404	2.00±0.14
44	IPI00229080	Hsp90ab1 Heat shock protein 84b	HSP90AB1	23	34	151	2.78±0.43
256	IPI00648105	Peroxiredoxin-1	PRDX1	11	61	327	2.00±0.18
290	IPI00759999	Peroxiredoxin-5	PRDX5	13	61	133	9.90±2.7
126	IPI00116154	Cytochrome c oxidase	COX5B	9	42	140	1.61±0.06

**Figure 5:**
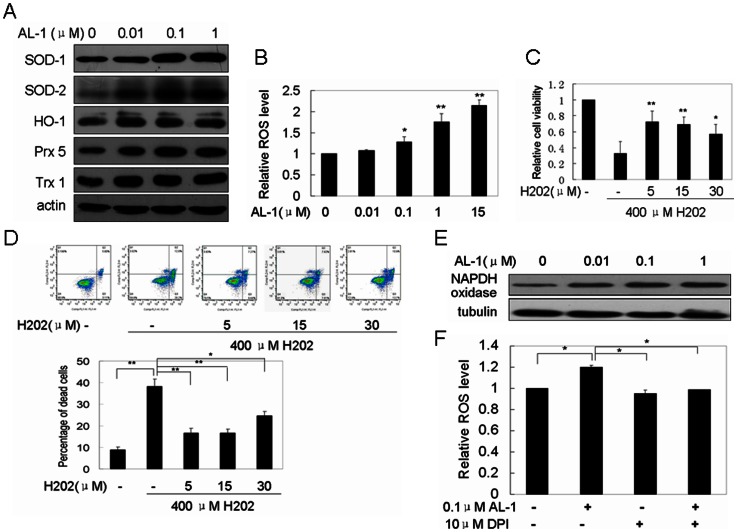
AL-1 upregulated NADPH oxidase, accompanying with the increases of ROS and the protein expression of antioxidation proteins Trx1, Prx5, HO-1, SOD1 and SOD2. (A) Antioxidation proteins Trx1, Prx5, HO-1, SOD1 and SOD2 were upregulated by AL-1 in a dose-dependent manner. (B) ROS level in RIN-mβ cells was increased by AL-1 in a dose-dependent manner. (C, D) Low dose H_2_O_2_ pretreatment also attenuated high dose H_2_O_2_-induced cell death. The cells were treated with the different concentration H_2_O_2_ (0, 5, 15, 30 μM) for 12 h prior to 400 μM H_2_O_2_ exposure for 4 h, the protective effects of AL-1 on H_2_O_2_-induced cell death were analyzed by MTT assay (C) and flow cytometer (D). (E) The NADPH oxidase expression was upregulated by AL-1 in a dose-dependent manner. (F) AL-1 stimulated ROS generation by upregulating NADPH oxidase. The RIN-mβ cells were pretreated with 0.1 μM AL-1 for 30 min, and then exposed to 10 μM NADPH oxidase inhibitor DPI for 30 min. The inhibition of NADPH oxidase blocked the AL-1-induced generation of ROS.

To validate whether AL-1 attenuated H_2_O_2_-induced cell death by regulating the expression of antioxidant proteins, some important antioxidant proteins including Trx 1, Prx 5, HO-1, SOD1 and SOD2 were selected for the expression analysis by Western blotting. As shown in Figure 5A, AL-1 upregulated the expression of antioxidant proteins Trx1, Prx 5, HO-1, SOD1 and SOD2 in a dose-dependent manner.

### ROS generation stimulated by AL-1

Previous studies have shown that the intracellular ROS could induce the expression of antioxidation proteins [22]. To determine whether AL-1 upregulated antioxidation proteins by generating ROS, the intracellular ROS level was analyzed after the RIN-mβ cells were exposed to the different concentrations (0.01, 0.1, 1, 15 μM) of AL-1 for 1 h. As shown in Figure 5B, AL-1 increased the intracellular ROS levels in a dose-dependent manner, suggesting that AL-1 stimulated the expression of antioxidation proteins by inducing ROS generation.

To identify that AL-1 attenuated high dose H_2_O_2_-induced cell death by generating low dose ROS, the cells were treated with low dose H_2_O_2_ with different concentrations (0, 5, 15, 30 μM) for 12 h prior to 400 μM H_2_O_2_ exposure for 4 h. MTT assay showed that the cell viability was increased when cells were pretreated with low dose H_2_O_2_ as compared to the treatment with high dose H_2_O_2_ alone (Fig. 5C). And the low dose H_2_O_2_ pretreatment decreased high dose H_2_O_2_-induced cell death (Fig. 5D).

### Upregulation of NADPH oxidase by AL-1 correlated with ROS generation

It is well known that NADPH oxidase is a major source of ROS in pancreatic β-cells [23], [24]. To determine whether AL-1 stimulated ROS generation by upregulating the expression of NADPH oxidase, the NADPH oxidase expression level was detected after the RIN-mβ cells were treated by AL-1. We found that the AL-1 treatment could significantly upregulate the expression of NADPH oxidase in a dose-dependent manner (Fig. 5E). To further validate that AL-1 stimulated ROS generation by upregulating NADPH oxidase, we used 10 μM NADPH oxidase inhibitor, diphenyleneiodonium (DPI), to suppress the activity of the AL-1-regulated NADPH oxidase as previously described [25]. As shown in Figure 5F, the pretreatment with DPI abolished the ROS generation induced by AL-1. Taken together, these results demonstrated that AL-1 increased the intracellular ROS level by upregulating the expression and activity of NADPH oxidase.

### Protein-protein interaction networks regulated by AL-1

STRING is a system for mapping protein-protein interaction networks. We used the system to construct the protein-protein interaction network of the differential proteins regulated by AL-1, showing that most of the 71 identified proteins can be mapped into a protein-protein interaction network (Fig. S4). Notably, MAPK (ERK1/2) and AKT1 were found to be the signal nodes in the network, suggesting that ERK1/2 and AKT1 signal pathways may be crucially involved in the anti-cytotoxic regulation of AL-1. This result is consistent with previous studies demonstrating that ROS and its regulator NADPH oxidase could activate ERK1/2 and AKT1 signaling pathways [25], and that ERK1/2 and AKT1 pathways played an important role in suppressing cellular apoptosis induced by H_2_O_2_ [26]–[28].

### AKT1 and ERK1/2 signaling pathways regulated by AL-1

**Figure 6:**
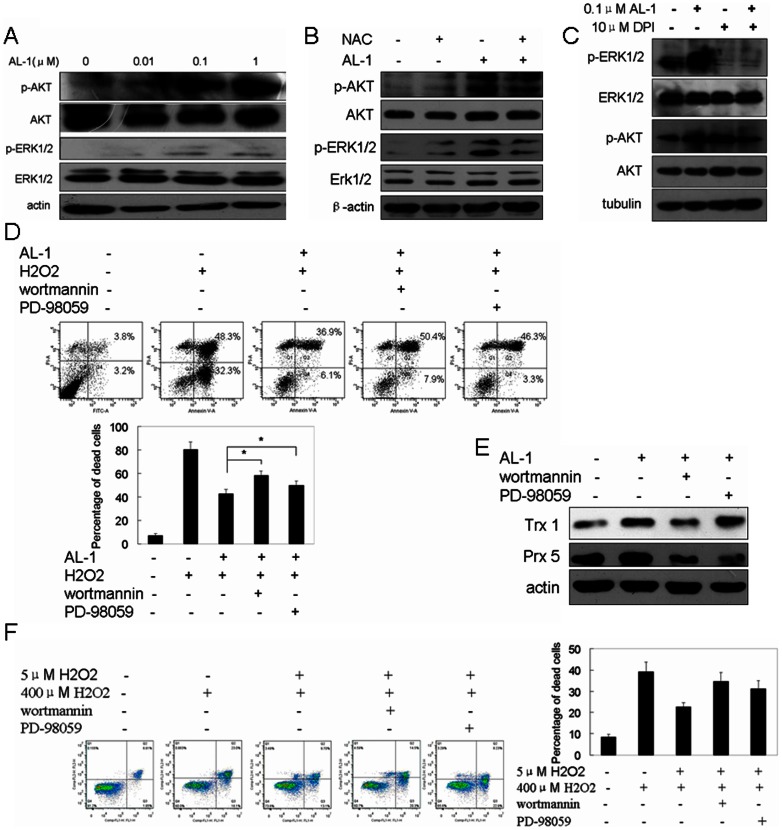
The AL-1-activated ERK1/2 and AKT1 signaling pathways were involved in the anti-H_2_O_2_-induced cell cytotoxicity of AL-1. (A) The phosphorylation levels of ERK1/2 and AKT1 were upregulated by AL-1 in a dose-dependent manner, with total ERK1/2 and AKT1 levels remaining unchanged. (B) ROS decrease by NAC treatment blocked AL-1-induced ATK1 and ERK1/2 activation. The AL-1-pretreated cells were further treated with NAC prior to H_2_O_2_ exposure, p-ERK1/2, ERK1/2, p-AKT and AKT levels were analyzed by Western blotting. (C) AL-1 increased the phosphorylation of ERK1/2 and AKT1 by upregulating NADPH oxidase. The RIN-mβ cells were pretreated with 0.1 μM AL-1 for 30 min, and then exposed to 10 μM NADPH oxidase inhibitor DPI for 30 min. (D) The anti-H_2_O_2_-induced cell death of AL-1 was mainly regulated by AL-1-activated ERK1/2 and AKT1. The cells were pretreated by combining 0.1 μM AL-1 with PI3K inhibitor wortmannin (250 nM) or the ERK1/2 inhibitor PD-98059 (25 μM) for 1 h, prior to the exposure to 400 μM H_2_O_2_ for 4 h. The number of apoptotic cells was analyzed by flow cytometer. The inhibition of ERK1/2 and AKT1 blocked the protective effect of AL-1 on H_2_O_2_-induced RIN-mβ cell death. (E) AL-1 upregulated the expression of antioxidant proteins Prx5 and Trx1 by activating AKT1 and ERK1/2 signaling pathways. (F) The protective effects of low dose H_2_O_2_ against high dose H_2_O_2_-induced cell death were also attenuated by AKT or ERK inhibition. The low dose H_2_O_2_-pretreated cells were further treated with AKT inhibitor wortmannin or ERK1/2 inhibitor PD-98059 prior to 400 μM H_2_O_2_ exposure, the cell viability was determined by flow cytometer.

The phosphorylation level at specific sites of protein kinases, including AKT1 and ERK1/2, represents their activity [29]. In order to validate that AL-1 could activate AKT1 and ERK1/2 signaling pathways by generating ROS, the phosphorylation and protein levels of AKT1 and ERK1/2 were analyzed in RIN-mβ cells treated with different concentrations of AL-1. As shown in Figure 6A, we found that the phosphorylation of ERK1/2 at Thr-202/Tyr-204 and AKT1 at Ser-473 was upregulated by AL-1 in a dose-dependent manner, with their total protein expression levels remaining unchanged. When AL-1-stimulated ROS generation was inhibited by antioxidant NAC, the AL-1-upregulated AKT1 and ERK1/2 phosphorylation was blocked (Fig. 6B). When AL-1-upregulated NADPH oxidase was inhibited by DP1, the activity of AKT1 and ERK1/2 was also repressed (Fig. 6C), suggesting that AL-1 activated ATK1 and ERK1/2 signaling pathways via NADPH oxidase-mediated ROS generation. The anti-H_2_O_2_-induced cell death of AL-1 was blocked when AL-1-activated ERK1/2 and AKT1 were inhibited by their inhibitors PD-98059 and wortmannin, respectively (Fig. 6D).

Intracellular ROS concentration was regulated by both free radical production and antioxidant defenses. The upregulation of antioxidant proteins can protect cell injury from oxidative stress by eliminating ROS [30], [31]. This could be the case that AL-1 protected cells from H_2_O_2_-induced cell cytotoxity by upregulating antioxidant proteins (Table 1 and Fig. 5A). To further confirm the correlation between the AL-1-activated AKT1 and ERK1/2 signaling pathways and antioxidant protein upregulation, we performed the inhibition experiments on AKT1 and ERK1/2 under AL-1 activation. As shown in Figure 6E, AKT1 inhibition decreased AL-1-upregulated Trx1 and Prx5 expression, while ERK1/2 inhibition blocked AL-1-induced Prx5 upregulation.

To further investigate that the protective effect of low concentration ROS was dependent on ERK1/2 and AKT1, the low dose H_2_O_2_-pretreated cells were further treated with AKT inhibitor wortmannin or ERK1/2 inhibitor PD-98059 prior to 400 μM H_2_O_2_ exposure, the cell viability was determined by flow cytometre. We found that the protective effects of low dose H_2_O_2_ against H_2_O_2_-induced cell death was attenuated by AKT or ERK inhibition, similar to the effects in AL-1 pretreatment (Fig. 6F).

**Figure 7:**
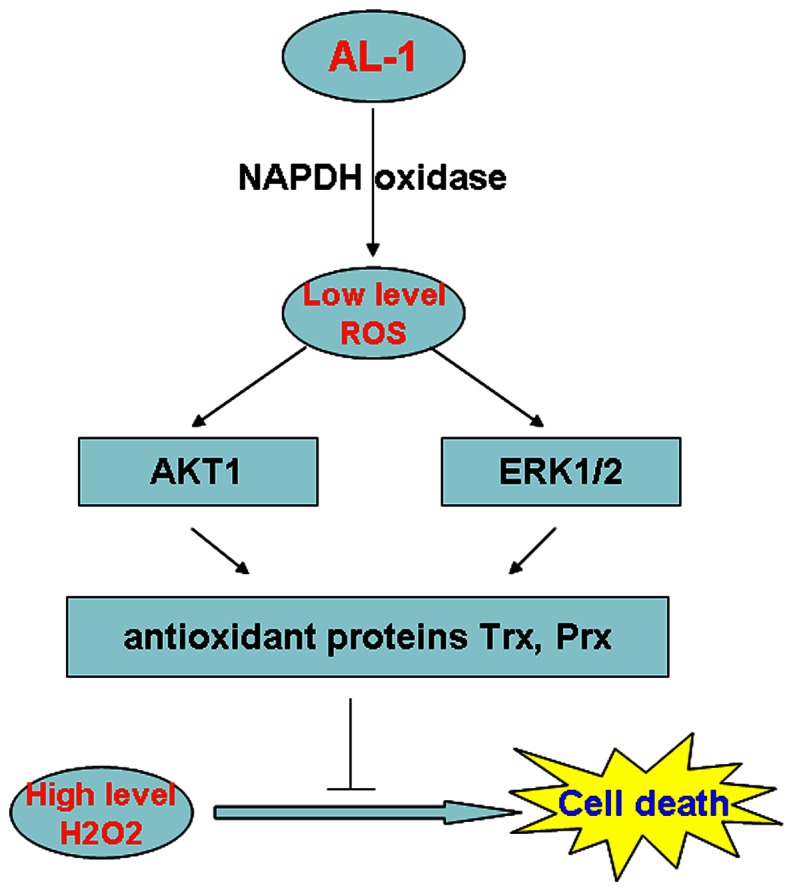
Molecular mode of the protective effects of AL-1 on H_2_O_2_-induced pancreatic β-cell death.

Taken together, these results suggest that AL-1 exerted its protective effects on H_2_O_2_-induced apoptosis by generating low dose of ROS to activate ERK1/2 and AKT1 signaling pathways and subsequently upregulated antioxidant proteins such as Trx1 and Prx5 (Fig. 7).

## Discussion

Oxidative stress has been implicated in a large number of human diseases such as diabetes, pulmonary fibrosis, atherosclerosis, cancer, cardiovascular disease, bronchopulmonary dysplasia, aging, and neurodegenerative disease. Some antioxidant nutrients and phytochemicals could alleviate oxidative stress-induced cell cytotoxity. Our previous studies showed that AL-1 could prevent ROS-mediated cellular injury; however, its molecular mechanism is still unknown. The novel finding in this study is that AL-1 can stimulate the generation of ROS in low concentration to work as messengers to activate ERK1/2 and AKT1 signaling pathways, subsequently upregulate antioxidant protein expression and then attenuate cell death induced by H_2_O_2_ with high concentration of ROS (Fig. 7). In the process, AL-1 induced the accumulation of ROS in β-cells by upregulating the expression and activity of NADPH oxidase (Fig. 5), consistent with the fact that NADPH oxidase is one of the main modulators in ROS generation in pancreatic β-cells [23], [32]. A novel molecular mechanism of antioxidation was here described, in which ROS with low concentration activated antioxidation system to prevent high concentration ROS-induced oxidative injury.

High concentration H_2_O_2_ in cells can result in apoptosis and necrosis by damaging cellular macromolecules, membranes and DNA, but ROS/H_2_O_2_ has also been considered as a ubiquitous intracellular messenger to regulate ERK1/2 and AKT1 signaling pathways at low concentration. The paradox in the roles of ROS as cellular messenger molecules in the regulation of cellular functions and as toxic by-products depends on whether its concentration is either below or above a specific threshold [33]. In this study, we demonstrated that AL-1 treatment with low concentrations (0.01, 0.1 µM) induced low concentration ROS generation in pancreatic β-cells, serving as signaling molecules to activate AKT1 and ERK1/2 signaling pathways and then upregulate antioxidant proteins (Figs. 5 and 6A). Reciprocally, we also found that AL-1 treatment with high concentration (5, 10, 15 µM) could induce leukemia K562 cell apoptosis by generating high concentration ROS [18]. This phenomenon resembles the function of nitric oxide (NO), which has both regulatory and cytotoxic effects depending on its relative concentration generated [33], [34]. NO functions as a secondary messenger to mediate vasodilation in low concentrations produced by the constitutive isoform of nitric oxide synthase (NOS) in vascular endothelial cells and also works as a source of highly toxic oxidants for microbicidal killing in high concentrations generated by inducible NOS in macrophages [33].

Increased ROS induced by some factors such as growth factor EGF played an important role in cell survival, proliferation, anti-apoptosis, invasion and metastasis, and angiogenesis in cancer cells [22]. ROS acted as a secondary messenger in growth factors-activated signaling pathways [35]. Here, we demonstrated for the first time that AL-1 could stimulate ROS generation and subsequently activate ERK1/2 and AKT1 signaling pathways. The AL-1-induced ERK1/2 and AKT1 phosphorylation was significantly blunted by pretreatment with antioxidant NAC and NADPH oxidase inhibitor (Figs. 6B, C), suggesting that the activation of NADPH oxidase is an important regulator of ERK1/2 and AKT1 in the setting of the anti-cytotoxicity of AL-1. In addition, the protective effects of AL-1 on H_2_O_2_-induced cell death were largely blocked by pretreatment with ERK1/2 and AKT1 inhibitors, PD-98059 and wortmannin, respectively (Fig. 6D), suggesting that the ERK1/2 and AKT1 activation was required for the AL-1-derived anti-apoptosis induced by H_2_O_2_.

The antioxidant proteins could protect cellular damage from high concentration ROS by catalyzing the reduction of H_2_O_2_ to H_2_O [33]. Loss of the antioxidant protein families, such as peroxiredoxins and thioredoxins, were associated with the accumulation of oxidatively damaged DNA [30]. Their overexpression could prevent cell injury from oxidative stress. For example, Prx-1 overexpression protected BECs from ROS-induced cell death [36]; induction of Trx1 expression protected the diabetic myocardium from dysfunction by reducing oxidative stress [31]. In this study, 13 antioxidant proteins, such as Prx1, Prx5, Trx1 and heat shock proteins were found to be upregulated by AL-1 treatment (Table 1). Subsequently, AL-1-induced overexpression of these antioxidant proteins could then protect RIN-mβ cells from H_2_O_2_-induced cell death.

ERK1/2 and AKT1 signaling pathways have been proven to upregulate the expression of antioxidant proteins Cu/Zn-SOD, Mn-SOD, HO-1 and HSP70 in oxidative stress [37]–[39]. The current observations showed that many antioxidant proteins were found to be upregulated by AL-1, and ERK1/2 and AKT1 were as connection hubs in the protein-protein interaction network containing the 71 AL-1-regulated proteins. The Prx6 and Trx 1 overexpression had been reported to activate AKT1 or/and EKR1/2 signaling pathways in lung cancer or ischemia [40], [41], we now showed in reverse that AL-1-activated AKT1 and ERK1/2 could increase the expression of Prx5 and Trx1 (Fig. 6E). In either way, the upregulated antioxidant proteins could be the source of messager ROS to stimulate the cellular defense system for the protection of cell damage.

In summary, we explored for the first time the molecular mechanism for the protective effect of AL-1 on H_2_O_2_-induced apoptosis by using proteomic analysis and follow-up functional characterizations. AL-1 exerted its anti-apoptotic effect by generating ROS as a signaling molecule to activate anti-apoptotic ERK1/2 and AKT1 signaling pathways and subsequently upregulate antioxidant proteins (Fig. 7). AL-1 worked as a “vaccinum” by generating low concentration ROS to activate the antioxidant system that then protected β-cell damage from ROS with high concentration.

## Materials and Methods

### Cell culture and reagents

RIN-mβ cell is an insulinoma cell line derived from a rat islet cell tumor [42]. Cells were purchased from the American Type Culture Collection and cultured in RPMI 1640 medium supplemented with 10% fetal bovine serum. AL-1 was synthesized and purified in the Institute of New Drug Research, College of Pharmacy, Jinan University, China [16]. MTT assay.

To measure the anti-H_2_O_2_-induced cytotoxicity of AL-1, MTT assay was performed in accordance with a previously reported procedure [19]. Briefly, the RIN-mβ cells were firstly treated with different concentrations of AL-1 (0.01, 0.1, 1 μM) for 1 h, and then exposed to 400 μM H_2_O_2_ for 4 h. In control groups, the RIN-mβ cells were only treated with 400 μM H_2_O_2_, 0.1 μM AL-1 or DMSO for 4 h, respectively. At the end of the treatments, the media were removed and the cells were stained and measured at 495 nm using an Autoplate reader (Bio-Tek, USA). The treatment with 400 μM H_2_O_2_ for 4 h could induce apoptosis of the half of RIN-mβ cells (49.8%). Therefore, 400 μM H_2_O_2_ treatment for 4 h was here selected as our oxidation model.

### Fluorescent staining of nuclei of AL-1 treated cells

Morphological changes in apoptosis process were analyzed as previously described with minor modifications [19]. In brief, 1×10^5^ RIN-mβ cells were firstly treated with 0.1 μM AL-1 for 1 h, and then treated with 400 μM H_2_O_2_ for 4 h. In control groups, the cells were only treated with 400 μM H_2_O_2_, 0.1 μM AL-1 or DMSO for 4 h, respectively. Cellular nuclear staining was then performed with Hoechst 33258, and cells were analyzed by a fluorescence microscope. The results were obtained through five independent experiments.

### Flow cytometric analysis of apoptosis

Cellular apoptosis was determined with flow cytometer as previously described [19]. For anti-H_2_O_2_-induced apoptosis of AL-1, cells were treated as stated above in Section 2.2. For anti-apoptosis of AL-1 by activating ERK1/2 and AKT1, the cells were pretreated by combining 0.1 μM AL-1 with 25 μM ERK1/2 inhibitor (PD-98059) or 250 nM PI3K inhibitor (wortmannin) for 1 h, followed by exposure with 400 μM H_2_O_2_ for 4 h. And these cells were then harvested, incubated with Annexin V-FITC, stained by propidium iodide (PI), and analyzed at 525 nm for FITC and at 630 nm for PI with a FACStar Plus flow cytometer.

### Western blotting

Cells were washed with ice-cold PBS three times and then lyzed as previously described [43]. Protein extracts were electrophoresed on SDS-PAGE gels and then electroblotted onto polyvinylidene fluoride membranes. The membranes were incubated with antibodies at 4°C overnight, respectively, followed by incubation with corresponding secondary antibodies. The antibody-bound proteins were detected by exposing to autoradiographic film.

### Two-dimensional electrophoresis (2-DE)

2-DE was performed as previously described [44]. The cells were lysed in lysis buffer (7 M urea, 2 M thiourea, 4% CHAPS, protease inhibitor). Proteins (150 μg) were subjected to IEF on 13 cm IPG strips, pH 3–10NL with Amersham Biosciences IPGphor IEF System (GE healthcare, Uppsala, Sweden). Samples were then transferred on to 12.5% SDS-PAGE for 2-D separation. The proteins in 2-DE gels were stained with silver. Each sample was analyzed three times. Images were scanned using an Image Scanner (GE Healthcare, Uppsala, Sweden), and semi-quantitatively analyzed using ImageMaster software. Only protein spots that were reproducibly different in all three experiments and significant (more than 1.5-fold) were selected for MS analysis.

### In-gel digestion and protein identification

Protein spots with differential expressions were in-gel digested as previously described with minor modifications [44]. Briefly, the differential protein spots were destained using 15 mM K_4_Fe(CN)_6_ and 50 mM sodium thiosulfate and digested with trypsin at 37°C overnight. Peptides were extracted from the gel spots. The extracted peptide solutions were dried in a SpeedVac centrifuge.

The peptide mixtures were analyzed on an ABI 4800-plus MALDI-TOF/TOF mass spectrometer (Applied Biosystems, Foster City, CA). The obtained MS and MS/MS data were processed by GPS Explorer software (V3.6) according to the default set, and peak lists were created, and proteins were then identified by the MASCOT search engine (V2.1) in IPI mouse database (V3.68) based on these MS and MS/MS spectra. Database searches were carried out using the following parameters: the trypsin enzyme was used; the error tolerance values of the precursor ions and the MS/MS ion masses were 50 ppm and 0.1 Da, respectively; and an allowance of two missed cleavages. Fixed modifications of carbamidomethyl (C) and variable modifications of oxidation (M) and were allowed. The MASCOT protein score of at least 65 was considered as statistical significance (p<0.05).

### Measurement of ROS

The intracellular ROS level was determined by DCFH-DA assay [45]. In brief, the RIN-mβ cells were treated with different concentrations of AL-1 (0.01, 0.1, 1, 15 μM) for 1 h, or the cells were pretreated with 0.1 μM AL-1 for 30 min and then exposed to 10 μM NADPH oxidase inhibitor DPI for 30 min as previously described with minor modifications [25]. These cells were then washed with serum-free RPMI1640 medium and incubated with DCFHDA at 37°C for 20 min. DCF fluorescence distribution of 1×10^4^ cells was detected by fluorospectrophotometer analysis.

### Protein categorization and protein-protein interaction analysis

The protein-protein interaction networks were mapped by the STRING (Search Tool for the Retrieval of Interacting Genes/Proteins) system as previously described [46]. The following sets of STRING were employed: organism, required confidence (score), interactions shown as “homo sapiens”, “medium confidence (0.400)”, “no more than 20 interactions”, and the other parameters were default settings.

### Statistical analysis

The Student's t-test was used for comparisons. Data are presented as mean ± SD. p<0.05 was considered to be significant.

## Supporting Information

Figure S1 **AL-1 had the protective effect when the pretreatment time was from 0.5** **h to 8** **h.** The cells were pretreated with 0.1 μM AL-1 for the different time prior to 400 μM H_2_O_2_ exposure for 4 h, the cell viability was analyzed by MTT assay.
(TIFF)

Figure S2 **The cell viability of co-treatment with AL-1 and H_2_O_2_ was not significantly different as compared to the treatment with H_2_O_2_ alone.** The cells were co-treated with the different concentration AL-1 (0, 0.01, 0.1, 1 μM) and 400 μM H_2_O_2_ for 4 h according to the reviewer's suggestion, the cell viability was determined by MTT assay.
(TIFF)

Figure S3 **Image overview of 2-DE gels for the proteins extracted from RIN-mβ cells pretreated with 0.1** **μM AL-1 for 1** **h and then exposed to 400** **μM H_2_O_2_ for 4** **h, and those treated with 400** **μM H_2_O_2_ only for 4** **h.** The proteins from RIN-mβ cells treated with and without AL-1 were separated by 2-DE, and the gels were stained with sliver. Shown are the representative results from three independent experiments.
(PDF)

Figure S4 **71 AL-1-regulated proteins were mainly involved in the ERK1/2 and AKT signaling pathways by STRING assay.**
(TIFF)

Table S1 **71 differential proteins regulated by AL-1 were identified by proteomics analysis.**
(PDF)

Table S2 **21 differential proteins were involved in anti-H_2_O_2_-induced apoptosis of AL-1 between AL-1+H_2_O_2_-treated cells and H_2_O_2_ alone-treated cells by proteomics analysis.**
(PDF)
