# Supplementary figures and images for: Protective Effects of Andrographolide Analogue AL-1 on ROS-Induced RIN-mβ Cell Death by Inducing ROS Generation
Source: PLoS One. 2013 Jun 4;8(6):e63656. doi: 10.1371/journal.pone.0063656 (PMC3672203; doi:10.1371/journal.pone.0063656)

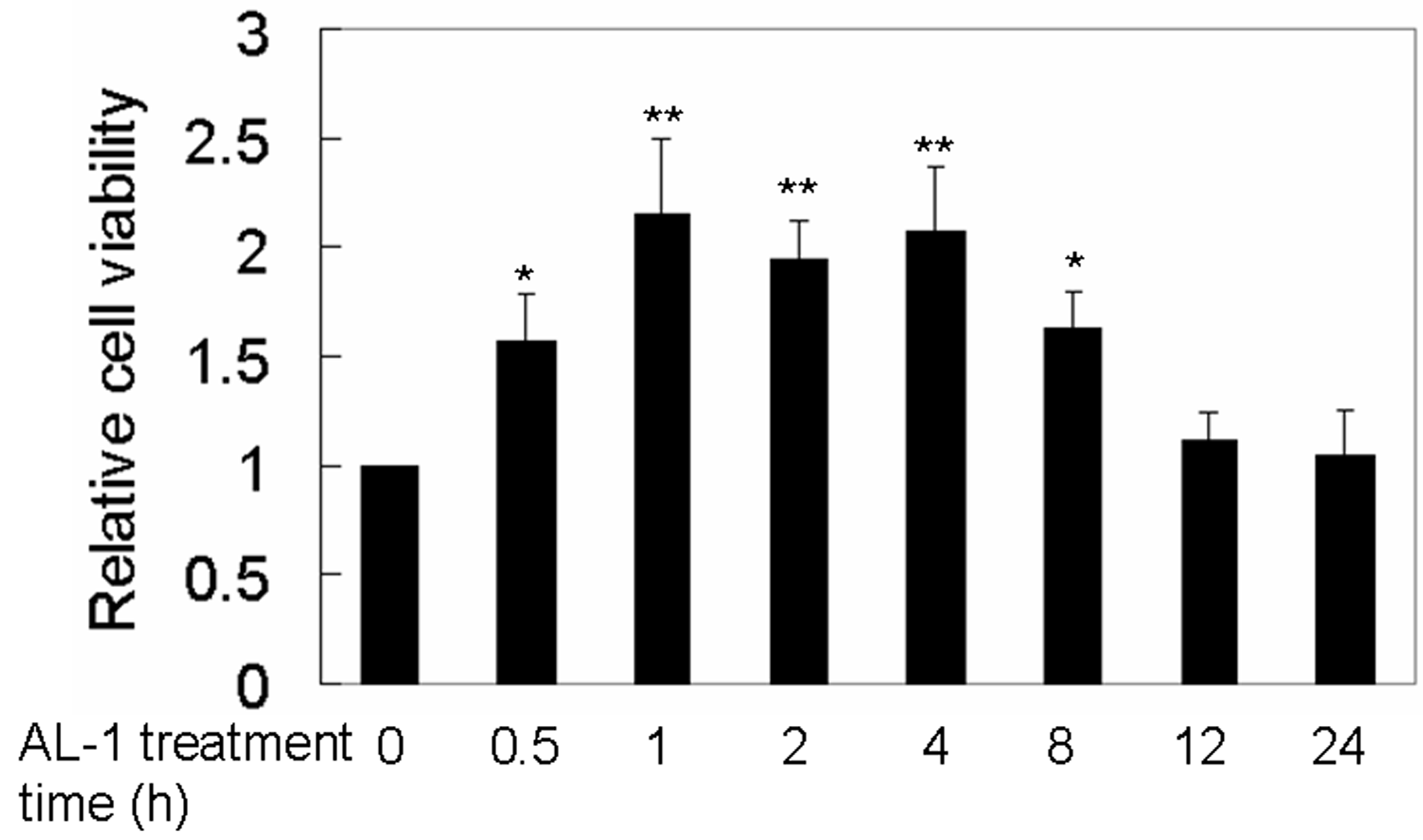

Supplement: Figure S1 — AL-1 had the protective effect when the pretreatment time was from 0.5 h to 8 h. The cells were pretreated with 0.1 μM AL-1 for the different time prior to 400 μM H2O2 exposure for 4 h, the cell viability was analyzed by MTT assay. (TIFF) [file pone.0063656.s001.tiff]

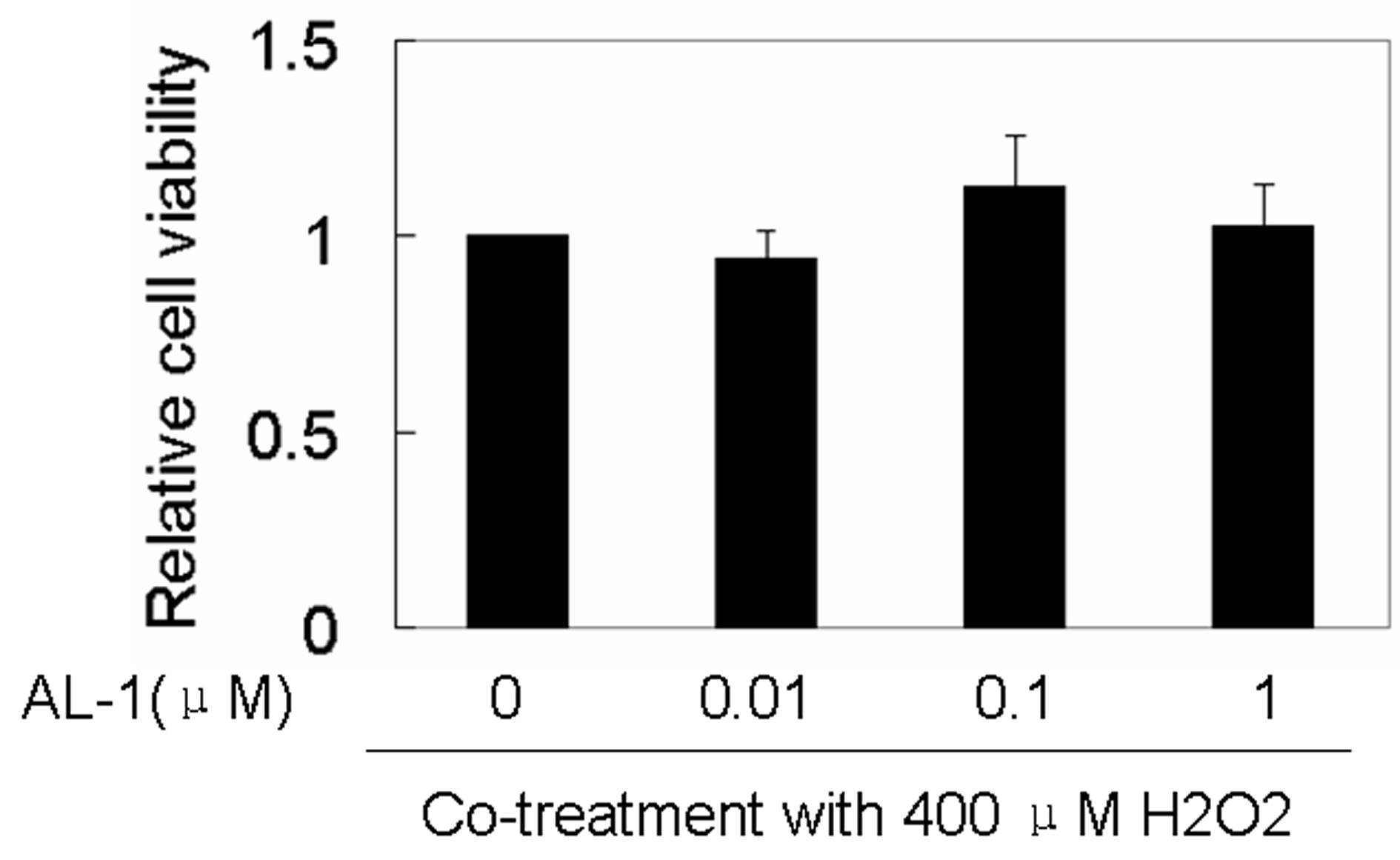

Supplement: Figure S2 — The cell viability of co-treatment with AL-1 and H2O2 was not significantly different as compared to the treatment with H2O2 alone. The cells were co-treated with the different concentration AL-1 (0, 0.01, 0.1, 1 μM) and 400 μM H2O2 for 4 h according to the reviewer's suggestion, the cell viability was determined by MTT assay. (TIFF) [file pone.0063656.s002.tiff]

### Supplementary Figure S3

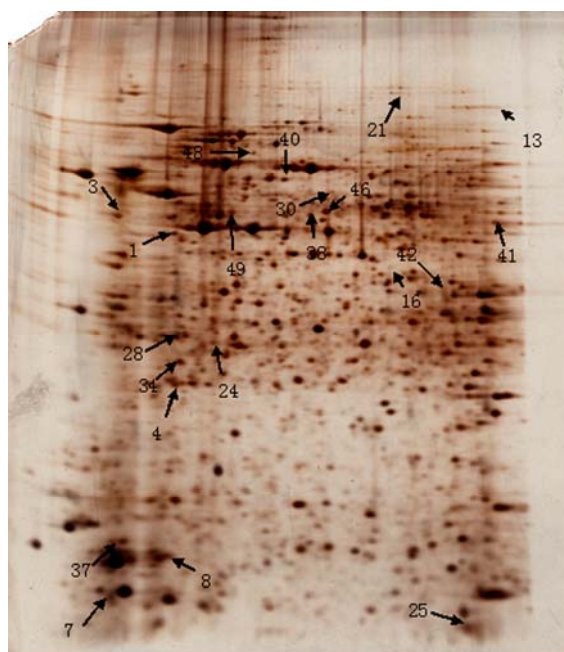

AL-1+H<sub>2</sub>O<sub>2</sub> treatment

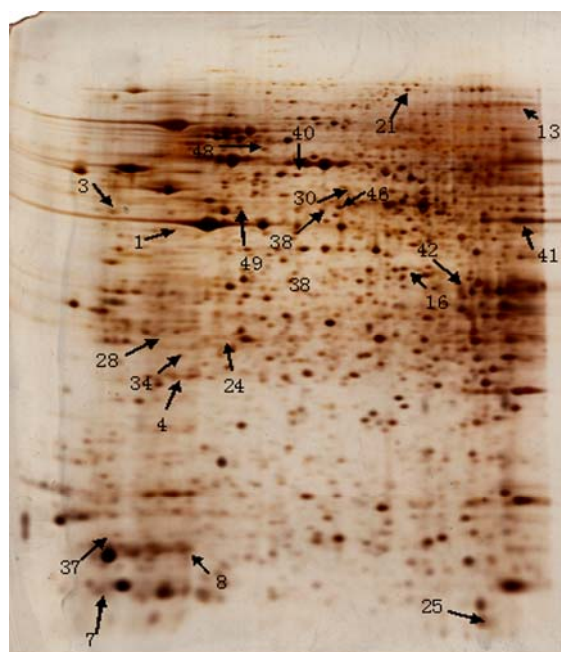

H<sub>2</sub>O<sub>2</sub> treatment

Supplement: Figure S3 — Image overview of 2-DE gels for the proteins extracted from RIN-mβ cells pretreated with 0.1 μM AL-1 for 1 h and then exposed to 400 μM H2O2 for 4 h, and those treated with 400 μM H2O2 only for 4 h. The proteins from RIN-mβ cells treated with and without AL-1 were separated by 2-DE, and the gels were stained with sliver. Shown are the representative results from three independent experiments. (PDF) [file pone.0063656.s003.pdf]

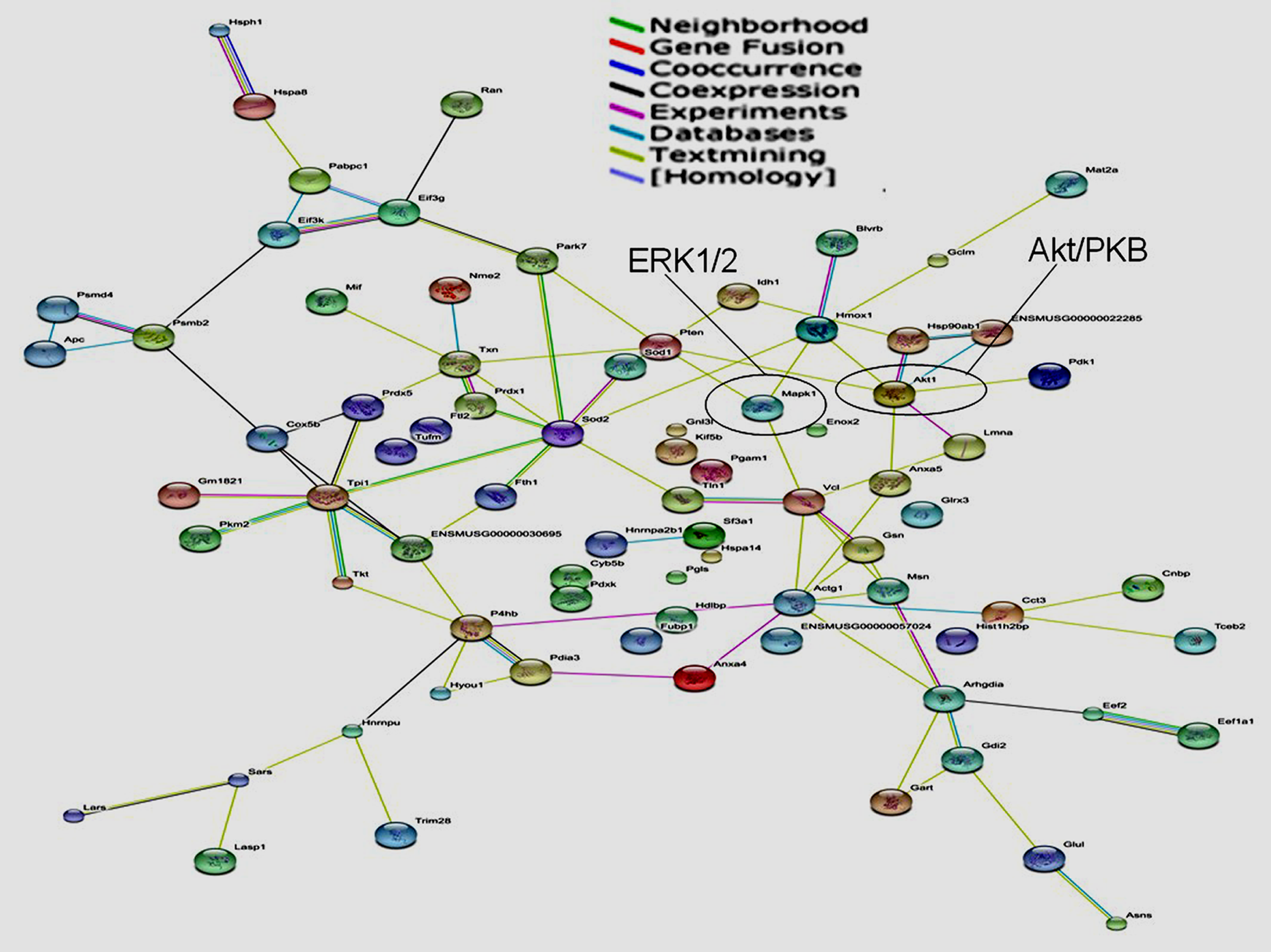

Supplement: Figure S4 — 71 AL-1-regulated proteins were mainly involved in the ERK1/2 and AKT signaling pathways by STRING assay. (TIFF) [file pone.0063656.s004.tiff]
