# Supplementary material for: Protective Effects of Andrographolide Analogue AL-1 on ROS-Induced RIN-mβ Cell Death by Inducing ROS Generation
Source: PLoS One. 2013 Jun 4;8(6):e63656. doi: 10.1371/journal.pone.0063656 (PMC3672203; doi:10.1371/journal.pone.0063656)
Supplement: Table S1 — 71 differential proteins regulated by AL-1 were identified by proteomics analysis. (PDF) [file pone.0063656.s005.pdf]

Supplementary Table S1. 71 differential proteins regulated by AL-1 were identified by proteomics analysis.

Spot No.	Accession No.	Protein Name	Gene Name	Theoretical MW(Da)	pI	Number of peptides	Coverage (%)	Protein Score	Ratio ± S.D.
276	IPI00467833	Triosephosphate isomerase	TPI1	26695.8	6.9	20	75	301	-2.9±0.3
40	IPI00132080	6-phosphogluconolactonase	PGLS	27237.4	5.55	16	58	661	2.84±0.4
294	IPI00137409	Transketolase	TKT	67587.6	7.23	17	37	251	-2.18±0.29
56	IPI00407130	Isoform M2 of Pyruvate kinase isozymes M1/M2	PKM2	57808	7.18	28	61	319	2.53±0.38
91	IPI00762452	isocitrate dehydrogenase 1 (NADP+), soluble	IDH1	46644.5	6.73	14	33	136	2.01±0.16
286	IPI00221402	Fructose-bisphosphate aldolase A	ALDOA	39331.3	8.31	25	71	328	-5.7±0.45
270	IPI00457898	Phosphoglycerate mutase 1	PGAM1	28813.9	6.67	16	56	210	-2.61±0.34
18	IPI00458583	Hnrnpu Osteoclast-like cell cDNA, RIKEN full-length enriched library, clon	HNRNPU	87862.7	5.92	10	12	195	4.07±0.21
48	IPI00312128	Isoform 1 of Transcription intermediary factor 1-beta	TRIM28	88790.5	5.52	22	25	92	2.6±0.38
80	IPI00131224	Transcription elongation factor B polypeptide 2	TCEB2	13161.7	4.87	9	92	205	2.17±0.13
76	IPI00331552	Pabpc1 Bone marrow macrophage cDNA, RIKEN full-length enriched library,	PABPC1	70625.9	9.52	24	31	104	2.25±0.125
298	IPI00330767	Isoform 1 of Cellular nucleic acid-binding protein	CNBP	19578.6	7.76	10	51	95	-10.5±3.37
51	IPI00408796	Splicing factor 3 subunit 1	SF3A1	88489.4	5.15	17	24	110	2.57±0.22
268	IPI00108818	Guanine nucleotide-binding protein-like 3-like protein	GNL3L	65153.5	8.85	17	29	68	-2.56±0.4
235	IPI00874931	Isoform 1 of Far upstream element-binding protein 1	FUBP1	68497	7.74	16	29	189	-1.6±0.16
282	IPI00127417	Nucleoside diphosphate kinase B	NME2	17351.9	6.97	6	44	85	-4.04±0.56
96	IPI00323644	Isoform Long of Trifunctional purine biosynthetic protein adenosine-3	GART	107327.6	6.25	20	26	158	1.89±0.08
57	IPI00405058	Isoform 3 of Heterogeneous nuclear ribonucleoproteins A2/B1	HNRNPA2B1	32440.4	8.74	15	58	134	2.52±0.23

64	IPI00466069	Elongation factor 2	EEF2	95252.9	6.41	35	39	416	2.408±0.25
69	IPI00120322	Isoform 1 of Eukaryotic translation initiation factor 3 subunit K	EIF3K	25070.4	4.81	10	44	320	2.34±0.24
242	IPI00116283	T-complex protein 1 subunit gamma	CCT3	60591.4	6.28	11	19	151	-1.63±0.18
61	IPI00230108	Protein disulfide-isomerase A3 precursor	PDIA3	56642.7	5.88	20	42	135	2.44±0.19
26	IPI00666161	similar to ribosomal protein S7 isoform 1	EG624124	22071.2	10.09	4	26	103	3.49±0.47
58	IPI00381291	Isoform Rpn10B of 26S proteasome non-ATPase regulatory subunit 4	PSMD4	41020.3	4.68	12	41	128	2.51±0.41
231	IPI00128945	Proteasome subunit beta type-2	PSMB2	22891.7	6.52	15	62	331	-1.56±0.17
24	IPI00307837	Eef1a1 Elongation factor 1-alpha 1	EEF1A1	50082.1	9.1	13	33	118	3.52±0.10
246	IPI00133522	Proteindisulfide-isomerase precursor	P4HB	57022.8	4.77	20	47	428	-1.77±0.23
10	IPI00274407	Isoform 1 of Elongation factor tu mitochondrial precursor	TUFM	49477	7.23	16	41	394	4.81±0.36
46	IPI00463886	similar to ribosomal protein L30	EG664969	12823.7	9.65	5	57	79	2.7±0.30
14	IPI00114329	Glutamate--cysteine ligase regulatory subunit	GCLM	30515.6	5.35	8	35	148	4.41±0.33
109	IPI00626366	similar to Acidic ribosomal phosphoprotein P0	EG667618	34194.8	5.91	20	55	379	1.77±0.26
259	IPI00277930	Capping protein	CAPG	38744.7	6.47	7	19	105	-2.16±0.5
284	IPI00116966	Asparagine synthetase	ASNS	64241.5	6.12	17	32	169	-4.46±0.48
124	IPI00124692	Transaldolase	TALDO1	37363.4	6.57	19	41	180	1.58±0.24
4	IPI00673707	similar to Bifunctional aminoacyl-tRNA synthetase	LOC633677	165528.3	8.15	34	29	123	7.11±1.69
2	IPI00321308	Alanyl-tRNA synthetase	AARS	106841.2	5.45	13	16	79	10.08±2.17
22	IPI00671695	phosphoribosylformylglycinamidine synthase	PFAS	144495.8	5.43	34	35	223	3.73±1.25
6	IPI00116498	14-3-3 protein zeta/delta	YWHAZ	27727.7	4.73	24	62	225	5.27±0.38
129	IPI00226993	Thioredoxin	TXN1	11667.6	4.8	9	57	128	1.76±0.15
7	IPI00416577	Isoform 2 of Rab GDP dissociation inhibitor beta	GDI2	46627.9	6.5	19	51	146	5.22±0.55
9	IPI00229894	Isoform 4 of Adenomatous polyposis coli protein	APC	296287.3	7.53	29	13	69	4.88±0.39

114	IPI00134621	GTP-binding nuclear protein Ran	RAN	24407.6	7.01	12	46	91	1.69±0.16
178	IPI00322440	Ubiquitin-conjugating enzyme E2-25 kDa	Ube2k	22392.6	5.33	11	43	210	1.83±0.18
34	IPI00405665	Isoform 1 of M-phase phosphoprotein 1	MPHOSPH1	203372.1	5.61	33	20	68	3.02±0.33
71	IPI00555125	Kif5b Kinesin family member 5B	KIF5B	109483.7	6.06	29	30	124	2.33±0.22
254	IPI00110588	Msn Moesin	MSN	67724.9	6.22	47	64	757	-1.94±0.36
15	IPI00465786	Tln1 Talin-1	TLN1	269665.1	5.82	41	20	199	4.31±0.35
55	IPI00405227	Vcl Vinculin	VCL	116644.3	5.77	22	24	98	2.54±0.23
32	IPI00759948	Isoform 2 of Gelsolin precursor	GSN	80712.4	5.52	19	34	205	3.13±0.33
257	IPI00317794	Nucleolin	Ncl	76676.8	4.69	20	26	177	-2.06±0.28
60	IPI00400300	Lmna Isoform A of Lamin-A/C	LMNA	65406.7	6.37	41	64	538	2.50±0.09
3	IPI00317309	Annexin A5	ANXA5	35730.2	4.83	18	42	175	7.27±1.14
134	IPI00877291	Annexin A4	ANXA4	35893.1	5.43	21	53	294	1.61±0.16
41	IPI00123379	Hdlbp Vigilin	HDLBP	141655.3	6.43	24	22	87	2.83±0.47
37	IPI00230139	FK506-binding protein 4	FKBP4	51539.9	5.54	18	44	125	2.87±0.28
127	IPI00116450	Isoform 1 of Core-binding factor subunit beta	CBFB	22016.8	5.59	13	51	100	1.63±0.15
175	IPI00153740	Activator of 90 kDa heat shock protein ATPase homolog 1	AHSA1	38093.2	5.41	14	42	200	1.56±0.05
49	IPI00123342	Hypoxia up-regulated protein 1 precursor	HYOU1	111112.2	5.12	27	32	225	2.64±0.26
89	IPI00323357	Hspa8 Heat shock cognate 71 kDa protein	HSPA8	70827.2	5.37	30	54	404	2.0±0.14
44	IPI00229080	Hsp90ab1 Heat shock protein 84b	HSP90AB1	83229.1	4.97	23	34	151	2.78±0.43
67	IPI00123313	Ubiquitin-like modifier-activating enzyme 1 X	Uba1	117734	5.43	22	25	132	2.36±0.20
256	IPI00648105	Peroxiredoxin-1	PRDX1	18914.7	6.82	11	61	327	2.0±0.18
250	IPI00230427	Macrophage migration inhibitory factor	MIF	12496.2	6.79	4	23	93	-1.85±0.23
290	IPI00759999	Peroxiredoxin-5	PRDX5	17004.1	7.71	13	61	133	9.9±2.7
277	IPI00625129	Ferritin light chain 2	FTL2	20725.5	5.66	11	64	243	-3.5±0.42

255	IPI00230145	Ferritin heavy chain	FTH1	21053.3	5.53	7	32	119	-1.99±0.22
23	IPI00315794	Cytochrome b5	CYB5B	16307.8	4.79	6	36	155	3.73±0.37
126	IPI00116154	Cytochrome c oxidase	COX5B	13837.9	8.34	9	42	140	1.61±0.06
62	IPI00875277	Hist1h2bk 14 kDa protein	HIST1H2BK	14184.7	10.24	10	58	125	2.42±0.44
291	IPI00113996	Flavin reductase	BLVRB	22183.4	6.49	6	33	93	-11.7±3.05
119	IPI00665601	similar to high-mobility group box 1	LOC637733	24591.1	6.43	11	41	101	1.61±0.21
