# Supplementary material for: Protective Effects of Andrographolide Analogue AL-1 on ROS-Induced RIN-mβ Cell Death by Inducing ROS Generation
Source: PLoS One. 2013 Jun 4;8(6):e63656. doi: 10.1371/journal.pone.0063656 (PMC3672203; doi:10.1371/journal.pone.0063656)
Supplement: Table S2 — 21 differential proteins were involved in anti-H2O2-induced apoptosis of AL-1 between AL-1+H2O2-treated cells and H2O2 alone-treated cells by proteomics analysis. (PDF) [file pone.0063656.s006.pdf]

Supplementary Table S2. 21 differential proteins were invovled in anti-H_2O_2-induced apoptosis of AL-1 between AL-1+H_2O_2-treated cells and only H_2O_2-treated cells by proteomics analysis.

Spot No.	Accession No.	Protein Name	Gene Name	Theoretical MW (Da)	pI	Number of peptides	Coverage (%)	Protein Score	Ratio ± S.D.
41	IPI00555069	Phosphoglycerate kinase 1	PGK1	44522	8.02	20	43	126	-2.32±0.33
13	IPI00116074	Aconitate hydratase, mitochondrial precursor	ACO2	85410	8.08	20	29	230	5.09±0.49
42	IPI00223757	Aldose reductase	AKR1B3	35709.4	6.71	10	33	67	-2.28±0.18
49	IPI00013847	ubiquinol-cytochrome c reductase core protein 1	UQCRC1	52612.4	5.94	11	14	191	-2.01±0.22
30	IPI00133916	Heterogeneous nuclear ribonucleo protein H	HNRPH1	49168.4	5.89	12	33	75	2.6±0.46
34	IPI00454008	Serine hydroxymethyltransferase	SHMT2	55725.6	8.72	18	33	94	2.48±0.247
1	IPI00468481	ATP synthase subunit beta	ATP5B	56265.5	5.19	18	32	259	3.59±0.30
24	IPI00133440	Prohibitin	PHB	29801.9	5.57	13	46	222	2.9±0.26
28	IPI00122815	17 days embryo kidney cDNA, RIKEN full-length enriched library, clone(P4hb)	P4HB	57022.8	4.77	15	29	75	2.69±0.15
25	IPI00649896	Ubc	UBB	25805.9	6.86	11	54	386	2.7±0.32
21	IPI00323483	Isoform 3 of Programmed cell death 6-interacting protein	PDCD6IP	96698.5	6.15	19	27	70	-3.4±0.44
8	IPI00263048	Nuclear mitotic apparatus protein 1	NUMA1	235515.4	5.68	35	16	69	-6.2±0.74
48	IPI00263048	Nuclear mitotic apparatus protein 1	NUMA1	235515.4	5.68	34	20	72	-2.02±0.08
4	IPI00322312	Rho GDP-dissociation inhibitor 1	ARHGDIA	23392.8	5.12	10	49	135	3.49±0.43
16	IPI00198567	LIM and SH3 domain protein 1	LASP1	29951.4	6.61	11	37	141	-4.54±0.77
38	IPI00400300	Isoform C of Lamin-A/C	LMNA	65406.7	6.37	26	44	120	2.41±0.24
40	IPI00880839	heat shock protein 9	HSPA9	73415.6	5.81	31	47	235	2.34±0.29
3	IPI00319992	78 kDa glucose-regulated protein precursor(Hspa5)	HSPA5	72377.5	5.07	25	39	346	3.9±0.55

46	IPI00308885	Isoform 1 of 60kDa heat shock protein,	HSPD1	60917.4	5.91	39	39	279	2.53±0.22
37	IPI00337844	E3 SUMO-protein ligase RanBP2	RANBP2	340876.8	5.82	36	13	60	2.42±0.30
7	IPI00224740	Profilin 1	PFN1	14947.5	8.46	7	67	61	5.23±1.1
